# A Sensor Array for the Detection and Discrimination of Methane and Other Environmental Pollutant Gases

**DOI:** 10.3390/s16081163

**Published:** 2016-07-25

**Authors:** Ami Hannon, Yijiang Lu, Jing Li, M. Meyyappan

**Affiliations:** 1 NASA Ames Research Center, Moffett Field, CA 94035, USA; ami.m.hannon@nasa.gov (A.H.); yijiang.lu-1@nasa.gov (Y.L.); jing.li-1@nasa.gov (J.L.); 2 Analtyical Mechanics Associates, Inc. at NASA Ames Research Center, Moffett Field, CA 94035, USA; 3 ELORET Corporation at NASA Ames Research Center, Moffett Field, CA 94035, USA

**Keywords:** gas sensor, room temperature gas sensing, functionalized nanotubes, principal component analysis, electronic nose, selective methane sensor, smartphone based sensor

## Abstract

We address the sensitive detection and discrimination of gases impacting the environment, such as CH_4_, NH_3_, SO_2_, and CO, using a sensor array and aided by principal component analysis (PCA). A 32-element chemiresistive sensor array consisting of nine different sensor materials including seven types of modified single-walled carbon nanotubes and two types of polymers has been constructed. PCA results demonstrate excellent discriminating ability of the chemiresistor sensor chip in the 1–30 ppm concentration range. The accuracy of the sensor was verified against data collected using cavity ring down spectroscopy. The sensor chip has also been integrated with a smartphone and has been shown to reproduce the sensing performance obtained with the laboratory measurement system.

## 1. Introduction

There is a great need for the development of sensitive, selective and stable sensors with a fast response for detecting various gases and chemical species in many industrial, medical, space exploration and environmental monitoring applications. Gases, such as CH_4_, CO, SO_2_, and NH_3_, are harmful either to the environment and/or to living beings and their monitoring requires sensors capable of detecting parts per million (ppm) level of these gases well below their OSHA permissible exposure limits. CH_4_ is the most important component of natural gas and the uncombusted methane can leak from the gas wells, pipelines or valves. When methane reaches 5%–15% by volume in air, the mixture is explosive. CH_4_ is also a greenhouse gas 20 times more detrimental than carbon dioxide. Hence, there is a need for a reliable, sensitive and selective sensor capable of detecting low concentrations of methane in real-time.

Metal oxide based sensors to detect methane and other environmental pollutants have been the subject of intensive research for several decades [1,2,3]. However, they generally work only at elevated temperatures and consume considerable amount of energy. Many studies have focused on carbon nanotube (CNT) based sensors to enable room temperature detection [4,5,6,7,8,9,10,11,12,13,14]. CNTs are promising as a sensing material due to their high surface area to volume ratio and readily available inner and outer walls for gas adsorption. Interaction of adsorbed gas molecules with CNTs can be specific which mostly results in some degree of charge transfer between the nanotubes and gas molecules. This interaction can be electron donating or withdrawing, which causes a change in conductivity of the CNTs [8]. The nanotubes as sensing materials have been mostly used in chemiresistor [12] or transistor [4,5] type devices. When there is no interaction between an analyte and pure CNTs, surface modification strategies such as doping, metal loading, functionalization, etc., are used to achieve desired selectivity and response to a specific gas. Functionalization of single wall carbon nanotubes (SWCNTs) with acid treatment has been well studied due to the need, not only in sensing, but also for applications in catalysis, composites, and many others [15,16,17,18,19,20,21,22]. A recent review of CNT-based gas sensors can be found in Reference [10].

The response of a given material (CNTs, graphene, conducting polymers, metal oxide thin films or nanowires) in terms of changes in resistance, capacitance or other physical properties for exposure to various gases has been well studied. Variations of these base materials with functional groups, doping, metal loading and other schemes have also been explored. However, the use of multiple materials in a sensor array and selective discrimination using pattern recognition has been rare with the use of emerging nanomaterials in sensor construction [5,6]. Here we report a 16–32 sensor array made with nine different sensing materials to achieve selective discrimination. We have used carboxylic SWCNTs, sulfonated SWCNTs, hydroxyl-functionalized SWCNTs and polyaniline to obtain room temperature detection of methane. We also used purified SWCNTs, polypyrrole, graphene, SWCNT functionalized with polyethylene glycol (PEG) and Pd doped SWCNT as additional sensing materials for the sensor array. We picked two polymers [23,24,25] in order to achieve a good degree of selectivity as the sensing mechanism for polymer-based sensors is different from that for CNT-based sensors [2,26,27]. Conducting polymers, such as polyaniline, polypyrrole and polythiophene, have been extensively used for fabrication of gas sensors, but, among them, polyaniline is the most common for the detection of various toxic gases [28,29,30,31,32].

The sensing materials presented in this work show capability of ppm level detection of CH_4_, CO, SO_2_, and NH_3_ at room temperature. Further, we also implement a pattern recognition technique known as Principal Component Analysis (PCA) to study the discrimination capability of the sensor array. The sensor data is compared against cavity ring down spectroscopy (CDRS) to establish the accuracy of our chemiresistive sensor. Finally, we demonstrate that a handheld device, such as a smartphone, can be used to detect CH_4_ gas at low ppm level at room temperature.

## 2. Experimental Section

**Sensing Materials Preparation.** Single-walled carbon nanotubes were purchased from Helix Material Solutions (Richardson, TX, USA). Chemical modification of SWCNTs was performed by acid treatment to obtain carboxylic-SWCNTs and sulfonated-SWCNTs as reported previously [17,18]. The SWCNTs were acid refluxed at 120 °C for about 4 h to allow sufficient time for reaction and achieve high level of carboxylic functionalization. Carbon nanotubes were also functionalized using potassium hydroxide to obtain hydroxyl (OH) functionalized SWCNTs [22]. These functionalization processes increase the surface activity and enable easier interaction with the target gases. The functionalized nanotubes were then dispersed in a solvent and sonicated for 2 h. Protonated polyaniline was used as another sensing material in the sensor array to help discriminating between various gases. Polyaniline base was protonated using strong hydrochloric acid to obtain a polyaniline salt [31]. The green precipitate was filtered, washed with distilled water and dried in a vacuum oven for 3 h at 40 °C. These four materials were synthesized specifically to achieve enhanced room temperature response to CH_4_. To enhance selectivity of the sensor array, we also used some commercially available materials, such as purified nanotubes (Carbon Nanomaterials Inc., Houston, TX, USA), polypyrrole (Sigma Aldrich, St. Louis, MO, USA), graphene (Sigma Aldrich, St. Louis, MO, USA), PEG-functionalized SWCNTs (Carbon Solutions, Inc., Riverside, CA, USA), and Pd-decorated SWNTs [32]. The choices made here for sensing materials are meant to be representative; additional and/or different materials from a range of possibilities including functionalized graphene, other two dimensional materials, nanoparticles and metal oxide nanowires can be used to provide wide chemical variations in constructing the multichannel sensor array chip.

**Sensor Fabrication.** The sensor array was fabricated using printed circuit board (PCB) as the substrate. The array consisted of 32 interdigitated electrodes, each with a finger gap size of 120 µm. All sensing materials were deposited and pipetted across the interdigitated electrode pattern of the sensor platform. Each material was deposited in three channels, as shown in Table S1. The evaporation of the solvent left a network of nanotubes on the electrodes to bridge the interdigitated fingers. The base resistance of the sensors was measured to be in the range of 500 ohm to 15 Kohm as shown in Table S1. The thickness of the film in each case was adjusted to get the desired base resistance.

**Gas Sensing Measurement.** The sensor chip was connected to an interface board allowing the measurement of individual resistances of each sensor using Keithley 2700 (Keithley Instruments, Inc., Scottsdale, AZ, USA). A computerized gas blending system, Environics 2000 (Environics Inc., Tolland, CT, USA) was used to create different gas concentration streams. Dry air was used both as a purge gas and a blending gas. A steady total flow of 400 cm^3^/min was used to introduce various gas streams to the sensors. The sensor chip was plugged into an electronic board and exposed to the gas stream using a Teflon cover placed right over the chip to prevent gas leaks into and out of the sensor system and to ensure correct gas concentration. All gas exposures were done after 10 min stabilization of baseline response in dry air (99.99% purity, Airgas Inc., San Jose, CA, USA) at room temperature. For measuring the sensor response, 2 min of target gas exposure was followed by a 5 min of dry air purge. To test various concentrations of the target gases, alternative air purge and target gas were introduced to the chip. CH_4_ (200 ppm, Airgas), NH_3_ (100 ppm, Airgas), SO_2_ (200 ppm, Airgas), and CO (200 ppm, Airgas) cylinders were used as source gases for the study. The sensor chip was exposed to these gases in a varying concentration range using the test set-up shown in Figure S1 under Supplementary Information. A Labview program was used to collect the sensor response data.

## 3. Results and Discussion

**Characterization of Sensor Elements.** Field emission scanning electron microscopy (FESEM) (Hitachi S-4800 FESEM) was used to study the morphologies of the sensing materials. The sensing material was deposited on a silicon substrate for obtaining SEM images (Figure S2, SI) instead of imaging the chip directly but the sensor elements were fabricated on PCB substrate as mentioned before. The pristine nanotubes have a uniform morphology and appear as separate nanotubes with 1–5 nm diameters and 0.1–3 µm lengths. All the functionalized nanotubes seem to be a tangled network of multiple nanotubes. The average diameter of both carboxylic and sulfonated SWCNT bundles appears to be about 5–10 nm and the length of the bundles is 0.1–1 µm. Both acid-treated SWCNTs seem to be rough and fragmented due to strong acid etching [33] and sonication. The sulfonated-SWCNTs are covered by a layer of foreign material, which can be groups of the sulfonic acids [19,34]. Potassium hydroxide-treated SWCNTs appear as thickened nanotubes and these alkali-treated nanotubes do not seem to be as damaged as acid-treated tubes. The hydroxyl-SWCNTs have uniform morphology and are not tangled to form a cluster. The average diameter of the bundle seems to be around 7–15 nm and the length of the tubes is 0.5–1 µm. Protonated polyaniline exhibits a homogeneous structure and the interaction between the particles is stronger. The polyaniline consists of porous networks and the connected particles are few microns long and about 800 nm in diameter. This polyaniline morphology is similar to that reported by other studies [35].

Infrared spectra were recorded at room temperature using Fourier transform infrared spectrometer (Perkin Elmer, San Jose, CA, USA) in the range of 400–4000 cm^−1^ with KBr pellets (see Figure S3 under Supplementary Information for representative spectra). Every wavelengh of light tranmitted in the spectra is characteristic of a specific chemical bond. The spectrum of carboxylic-SWCNTs shows peaks at 1723 cm^−1^, which can be assigned to the C=O strength vibration in COOH group. The peak at 1590 cm^−1^ is graphite-like E1u mode, also known as G band, and originates from the SP^2^-hybridized carbon [36,37]. Another band seen in Figure S3a is a very broad stretching O-H peak at 3450 cm^−1^. The O-H peak is more pronounced in acid-treated nanotubes due to the presence of more O-H groups from increased carboxylic groups. The peak observed at 1384 cm^−1^ comes from the vibration of the carboxylic group. The band at about 1145 cm^−1^ is assigned to C-O bonds. The intense peak at 743 cm^−1^ is attributed to the aromatic C-H out-of-plane deformation vibration. In the spectra of sulfonated-SWCNTs (Figure S3b), the stretching modes of the sulfate groups can be identified [19] with the peaks at 1385 and 1090 cm^−1^. The peaks at 658 cm^−1^ and 520 cm^−1^ can be assigned to the S=O stretching mode of -SO_3_H and C-S stretching mode, respectively [38]. The broad peak in the region of 2990–3700 cm^−1^ shows the presence of O-H groups. The FTIR spectra in Figure S3c of the hydroxyl-SWCNTs show a broad peak at ~3460 cm^−1^, which is a characteristic of the hydrogen bonded O-H stretch of hydroxyl group [22,39]. The band at 1372 cm^−1^ can be interpreted as the bending stretching band of the hydroxyl groups [40]. The strong transmission band at about 1591 cm^−1^ can be attributed to the C=C stretching mode of the SWCNT graphitic structure. The FTIR results confirm that the hydroxyl groups have been introduced onto the SWCNTs.

In Figure S3d, for the polyaniline, the band observed at 3435 cm^−1^ is due to the N-H stretching vibrations. IR spectroscopy can distinguish between the benzenoid rings and quinoid rings in the 1300–1600 cm^−1^ region of the spectrum. The bands near 1600 cm^−1^ and 1453 cm^−1^ are assigned to the nonsymmetric C6 ring stretching modes of quinoid and benzenoid ring vibrations, respectively [41]. The absorption band at 1290 cm^−1^ is assigned to C-N stretching of secondary aromatic amines [41,42]. The band at 1118^−1^ is a measure of the degree of electron delocalization and hence it is a characteristic peak of polyaniline conductivity [43]. The polymers show two intense bands at 1120 and 620 cm^−1^, representing in plane and out of plane C-H bending motions of benzenoid rings. The stretching bands that are characteristics of an aromatic amine are observed in the region between 1230 and 1350 cm^−1^. The peak seen at 2938 cm^−1^ can be assigned to C-N stretching of secondary aromatic amine [44].

**Figure 1:**
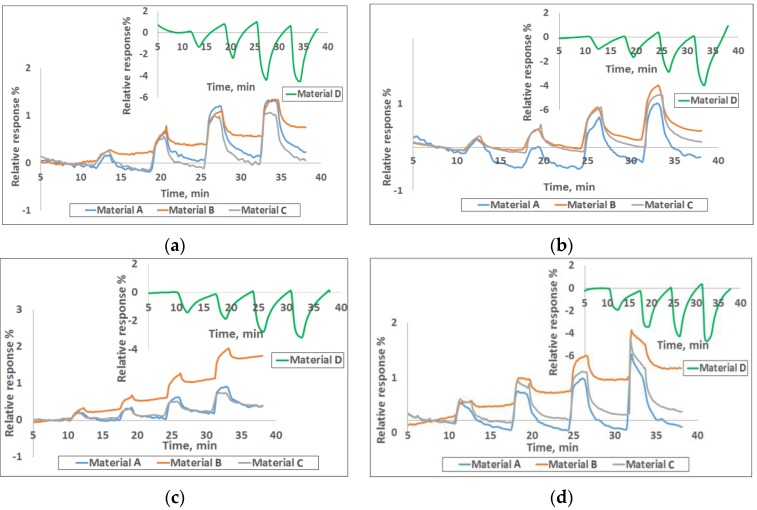
Response of various sensor materials to (**a**) 2, 5, 10, 25 ppm CH_4_; (**b**) 2, 7, 17, 27 ppm CO; (**c**) 2, 5, 12, 27 ppm SO_2_ and (**d**) 2, 5, 10, 30 ppm NH_3_. Materials A, B, C and D represent carboxylic-SWCNTs, sulfonated-SWCNTs, hydroxyl-SWCNTs, and polyaniline.

**Figure 2:**
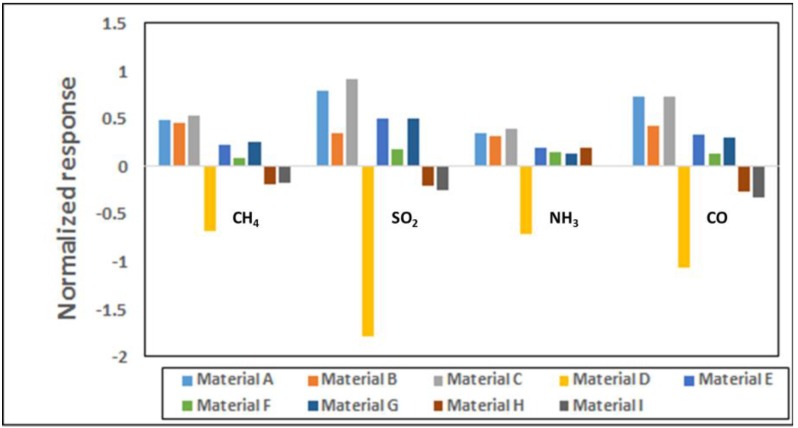
Normalized reponse in bar chart form for 25 ppm concentration of target gases CH_4_, SO_2_, NH_3_ and CO. The error bars range from 1.5% to 6.0%. The nine materials are listed in Table S1.

**Sensing Results.** The sensor chip was exposed to CH_4_, CO, SO_2_, and NH_3_ in the concentration range of 1–30 ppm at the interval shown in Figure 1. The corresponding calibration curves providing the response vs. concentration are given in Figure S4. The sensors were purged with an airflow rate of 400 cm^3^/min for the first 10 min, as well as before and after any target gas exposures. All the tests were performed at room temperature. Figure 1 shows the response curves for four of the nine materials to various concentrations of the target gases. The response plotted here is the normalized resistance (*R* − *R*_0_)/*R*_0_, where *R*_0_ is the baseline resistance before gas exposure and *R* is the instantaneous resistance at any time t after the gas exposure. It is well known that chemiresistive sensors generally drift with time and therefore, signal processing is done by looking at the relative change in the slope of the response curve when gas exposure occurs. The conductivity change of the sensors is concentration dependent and it increases or decreases linearly with gas concentration in the range of 1–30 ppm for various gases in a unique manner. Sensor responses to various gases depend on both the chemical nature of the sensing material and the target gas. The electrical resistance of the three SWCNT-based materials in Figure 1 increased, while it decreased for polyaniline upon exposure to various target gases. Figure 2 shows the normalized responses in a bar chart for all nine materials for 25 ppm each of CH_4_, CO, SO_2_ and NH_3_ exposures. Polyaniline provides a much stronger response than the SWCNT based materials for all gases; however, our experience indicates performance degradation over time for polymer based materials whereas the CNTs are more stable over longer periods. Within the observed response region in Figure 2, the three SWCNT-based candidates from Figure 1 show distinct responses for each of the four analytes. Though each material is used in three sensor channels of the sensor array (Table S1), the sensor-to-sensor variation among the three can be 5-10% based on extensive characterization in our lab for various material-gas combinations. This is largely due to the manual drop-casting of the sensing material onto the chip and this variability is expected to go down when automated ink-jetting or an equivalent process is used.

**Sensing Mechanism.** Carboxylic-SWCNTs treated in HNO_3_:H_2_SO_4_ mixture for 2 h did not show significant response to CH_4_ (data not shown here). Treatment lasting longer times (~4 h) yielded much improved response to CH_4_. It is possible that the longer acid treatment might have introduced more number of defects and carboxylic groups, allowing better sensitivity to the target gases. The presence of oxygenated functionalities at the ends of the SWCNTs facilitates electron transfer with target gases [16,45]. The larger response with oxygenated carbon nanotubes might be the result of introduction of more controlled carboxylic groups, which forms low-energy adsorption sites and facilitates charge transfer at defect sites [46]. Similar results were observed and reported with sulfonated SWNTs [19]. The change in conductivity resulting from interaction of certain gases with functionalized-SWCNTs has also been ascribed to the formation of hydrogen bond between the functionalized group and the target gas molecule. Matranga and Bockrath [47] and Dong et al. [48] reported the interaction between carbon monoxide and hydroxyl and carboxyl modified CNTs to be due to hydrogen bond formation. Similar to carboxylic-SWCNTs, sulfonic acid defects and hydroxyl defects also form low energy adsorption sites and facilitate charge transfer at defect sites [19,22]. Sulfonic acid group (SO_3_H) is even more acidic than the carboxylic group.

As mentioned earlier, all three functionalized SWCNTs in Figure 1 exhibit an increase in resistance upon exposure to CH_4_, CO, SO_2_, and NH_3_ gases while polyaniline shows the opposite behavior. Doped polyaniline is widely used to detect acidic and basic gases and the conductivity of polyaniline depends on both the oxidation state of the main polymer chain and the degree of protonation on imine sites [49]. Any interaction with polyaniline that alters either of these two statuses will affect its conductivity. Interaction between polyaniline and the adsorbate may cause further doping/de-doping of the polymer or cause swelling of the polymer and thus, lead to an increase or decrease in electrical conductivity [24,50]. When polyaniline interacts with CH_4_, CO, SO_2_, and NH_3_, these gases behave as reducing agents to donate electrons to polyaniline, causing a decrease in resistance of the sensor film. This indicates that protonated polyaniline is N-type doped. In some cases, the polymer-target gas interaction can be attributed to hydrogen bonding as well as dipole-dipole interactions [51]. Additional thorough investigations are needed to state the exact sensing mechanism with various target gases. The reversibility of these reactions makes the conductive polymer materials particularly useful for gas sensing applications.

**Figure 3:**
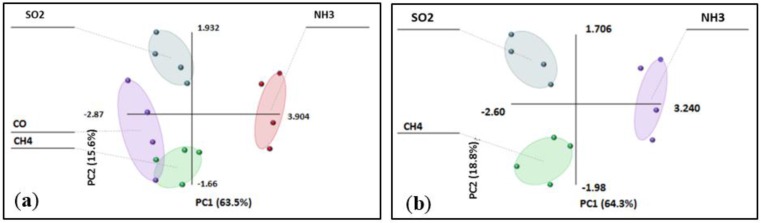
Principal component analysis of sensor array response to (**a**) CH_4_, CO, SO_2_ and NH_3_; (**b**) CH_4_, SO_2_ and NH_3_.

**PCA Study.** After exposing the nine different materials to various gases, the sensor discrimination ability was studied using principal component analysis, a technique widely used in electronic nose data analysis to discriminate gases/vapors. The analysis expresses the main information in the variables by lowering the number of variables, the so-called principal components. It is an orthogonal projection of data from a higher dimensional space to a lower dimensional one so that the variance of the projected data is maximized. In our case, the data matrix was constructed with the rows representing the responses to a gas from different sensing materials and the columns representing the response of each sensing material to different gases at two different concentrations (15 and 25 ppm). Figure 3a shows that each gas clusters together displaying a clear distance between them except that the CO cluster interferes with CH_4_. Figure 3b shows that CH_4_ can be easily discriminated from SO_2_ and NH_3_. Further discrimination between CH_4_ and CO would require materials, which behave differently to these gases.

**Figure 4:**
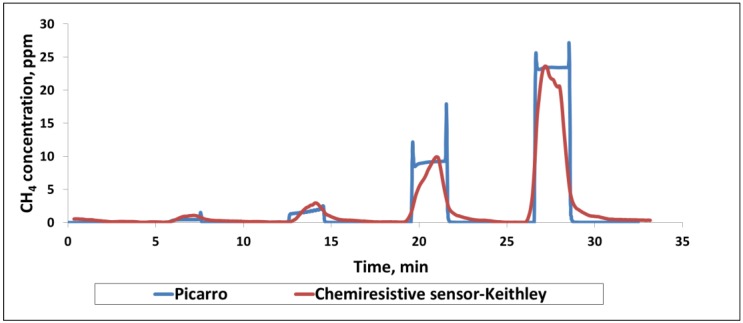
Picarro CRDS and chemiresistive sensor responses from the Keithley instrument for sufonated nanotubes to various ppm level concentrations of CH_4_.

**Comparison with Analytical Instrument.** One of the goals in sensor development is to achieve sensitive and selective detection of target gases with sufficient accuracy so that the sensor can replace the more established and well known—but bulky and expensive—instruments. Therefore, it is important to establish the accuracy of sensor data by comparing against data obtained from a known instrument. Cavity ring down spectroscopy (CRDS) is very accurate and provides time dependent measurements using a laser for quantifying the spectral features of gas phase constituents. Here, we used a Picarro Model G2301 (Figure S5) as a gold standard to compare our sensor performance. This instrument has been previously flown in NASA unmanned aerial vehicles (UAVs) in earth monitoring missions. It measures CO_2_, CH_4_ and water vapor with negligible long term drifts (months). The unit is approximately 43 × 18 × 45 cm in size and weighs ~27 Kg including an external vacuum pump, and it would be ideal to replace it with a small sensor such as the one discussed in this work for use in earth observation missions. As shown in Figure S6, the Environics gas dilution system produced the targeted concentrations and then a T-valve distributed the flow into two equal streams of 400 cc^3^/min. One gas flow stream entered into the Picarro CRDS system and other through the chemiresistive sensor chip. Both the chemiresistive sensor and the Picarro CRDS system responded well to CH_4_ in the concentration range of 1–30 ppm at room temperature (Figure 4). Only the sensor data from the sulfonated-SWCNTs of the chemiresistive chip are shown here for convenience. Picarro’s response and recovery are relatively instantaneous, matching the step change in concentration corresponding to the turning on and off of the methane supply. In contrast, chemiresistive sensors take 30–60 s to respond to methane. As a result, the shapes of the response vs. time curve do not match exactly. However, the predictive capability of the chemiresistive sensor and its accuracy are very close to those of the instrument.

**Figure 5:**
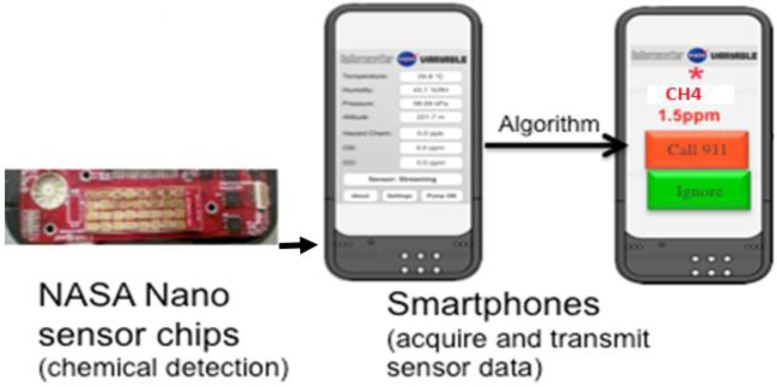
Phone sensor module and its integration with smart phone. The unit on the left contains the sensor chip on PCB and a microfan on the upper left corner. This unit can be attached at the bottom of the phone using the pins. The device can also be configured as a Bluetooth module as well.

**Figure 6:**
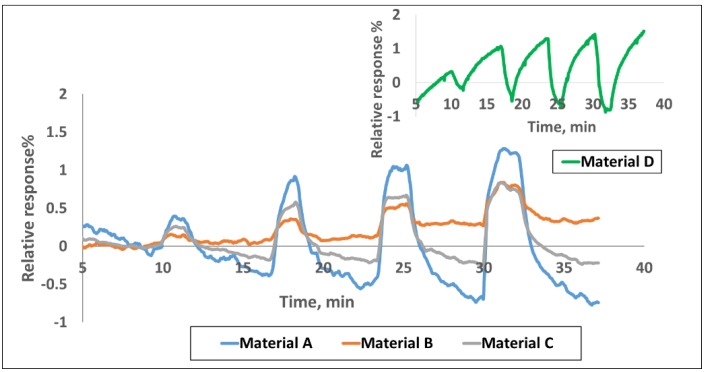
Responses of various sensor materials to 2, 5, 10, 25 ppm CH_4_ using the smartphone sensor module.

**Smartphone Sensor Module**. The chemiresistive sensor array was tested for methane detection on a smartphone sensor device (Figure 5). Integration of the sensor chip with a mobile phone has many advantages such as low cost platform, compactness, low power consumption, easy operation and rapid sensing capability in a mobile environment for various applications. The experimental set-up used for this test is similar to the one shown in Figure S1 except that the miniaturized phone sensing module takes care of the current-voltage measurement and data conversion instead of the Keithley system in the laboratory. The sensing module consists of the 32 sensor chip on PCB substrate with four materials (same as the one tested in the laboratory with the Keithley system) and an optional microfan. The small holes on the top of the unit allow the ambient air to reach the sensor chip. The module, approximately 2.5 cm × 3.8 cm in size, can be plugged into the phone as shown in Figure 5. The smart phone’s data processing capability is used for data acquisition, storage and processing. The phone-based sensor response for various concentrations of methane is shown in Figure 6 for the four materials represented in Figure 1. The corresponding calibration curves are shown in Figure S7; these curves were obtained by excel equation fitting by adding a trend line and checking for the regression model that shows the highest fit (*R*^2^ closest to 100%). The target gas duration was 2 min. each and the air purge was 5 min in the beginning and 5 min between exposures. The results generally match well with the data obtained with the Keithley system. However, quantitative comparison of the normalized response (*R* − *R*_0_)/*R*_0_ between the two systems is not possible because the Keithley instrument uses 22 bit resolution and the iPhone uses only 12-bit resolution. This difference in resolution general does not matter when a given material-gas combination yields a pronounced response, for example chlorine with some of the functionalized nanotubes from our previous experience; however, methane response here is ~2% for all the materials. More important is the predictive capability in terms of identification and gas concentration, and that is not impacted.

## 4. Conclusions

A gas sensor array was developed on a PCB based sensor chip with nine different sensing materials including various functionalized SWCNTs and polymers. The sensor array was tested for sensitivity to CH_4_, CO, NH_3_ and SO_2_. The array data showed difference in sensing behavior and produced a unique response for different combinations of sensing material and target gas. The PCA exercise confirmed the presence of definite patterns in sensor responses and as a result, the ability to discriminate CH_4_ from SO_2_ and NH_3_. CO interferes with CH_4_ detection and we need to focus on synthesizing new materials that respond very differently to these two gases in order to achieve discrimination between them. The sensor chip has been compared with a conventional instrument and it shows similar methane detection capability with advantages of smaller size, low power and low cost. We have also demonstrated CH_4_ detection at low ppm using a smartphone sensing device. The integrated phone can be used for applications where real time, in situ measurement of trace chemicals is required. The advantages of the phone sensor combined with the sensor discrimination power open up an opportunity to develop a nanosensor array for E-nose application for toxic gas detection, chemical leak detection, process control, and other military, industry, medical and space applications. Sensor technologies suitable for deploying in real world applications require additional features such as ruggedness, reliability, long term stability, sensor refreshing protocols and understanding and accounting for the environmental impact [10]. For example, we have previously considered the effect of humidity as an interferant in NO_2_ sensing [52] and found that it affects the sensor response requiring response curves of the type in Figure 1 and calibration curves to be generated for multiple humidity levels for later field use in interpolation and PCA. The demonstration in this work represents additional steps towards realizing the potential of long investigated CNT based sensors for gas/vapor detection.

## Supplementary Materials

This supplementary information consists of the following information: base resistance of each material in the sensor array; schematic of the experimental set-up used for sensor testing as well as for comparison against the Picarro CRDS instrument; SEM images of various functionalized SWCNTs; FTIR characterization of various functionalized SWCNTs; and calibration curves showing response vs. concentration for the sensor chips and the smartphone sensor. The following are available online at http://www.mdpi.com/1424-8220/16/8/1163/s1.
