# Supplementary material for: A Sensor Array for the Detection and Discrimination of Methane and Other Environmental Pollutant Gases
Source: Sensors (Basel). 2016 Jul 25;16(8):1163. doi: 10.3390/s16081163 (PMC5017329; doi:10.3390/s16081163)
Supplement: Supplementary file 1 [file sensors-16-01163-s001.pdf]

Supplementary Materials: A Sensor Array for the Detection and Discrimination of Methane and Other Environmental Pollutant Gases

Ami Hannon, Yijiang Lu, Jing Li and M. Meyyappan

Table S1. Sensing materials used in each sensor channel and the corresponding base resistance.

	Material Type	Designation	Average Resistance (K·Ω)
1–3	Carboxylic-SWCNTs	Material A	1.63
4–6	Sulfonated-SWCNTs	Material B	19.8
7–9	Hydroxyl-SWCNTs	Material C	0.98
10–12	Polyaniline	Material D	13.6
13–15	Purified-SWCNTs	Material E	0.22
16–18	Polypyrrole	Material F	2.25
19–21	Graphene	Material G	8.76
25–27	PEG-SWCNTs	Material H	14.7
28–30	Pd-SWCNTs	Material I	133.1

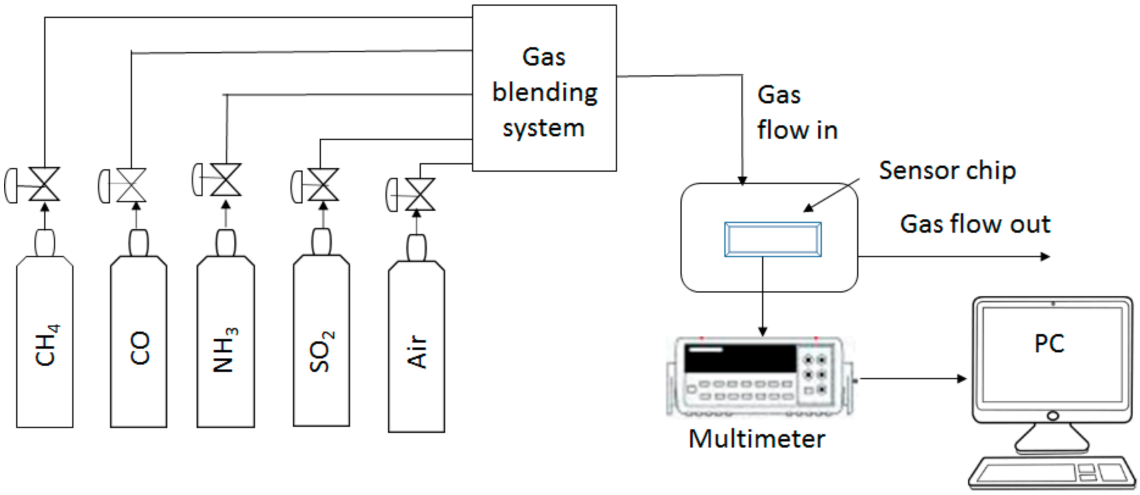

Figure S1. Schematic of the experimental set-up used for sensor testing.

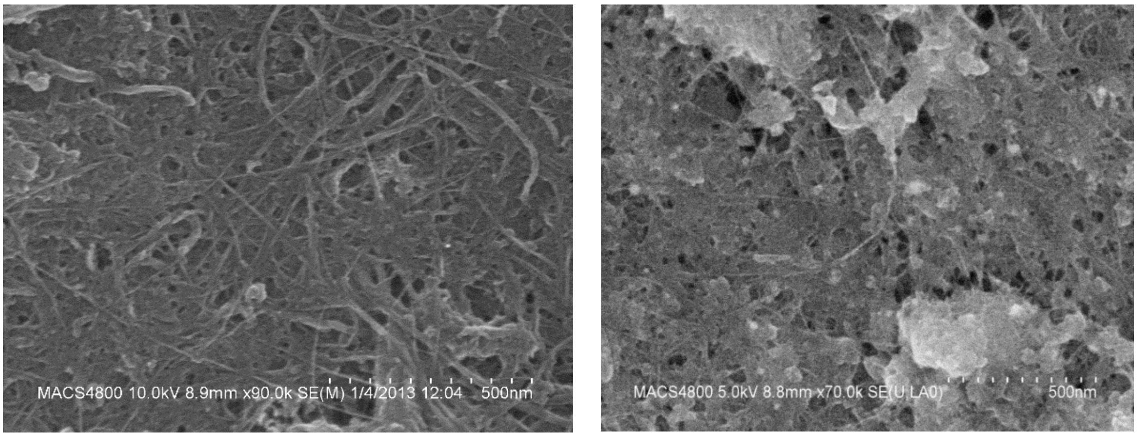

(**a**) (**b**)

Figure S2. *Cont.*

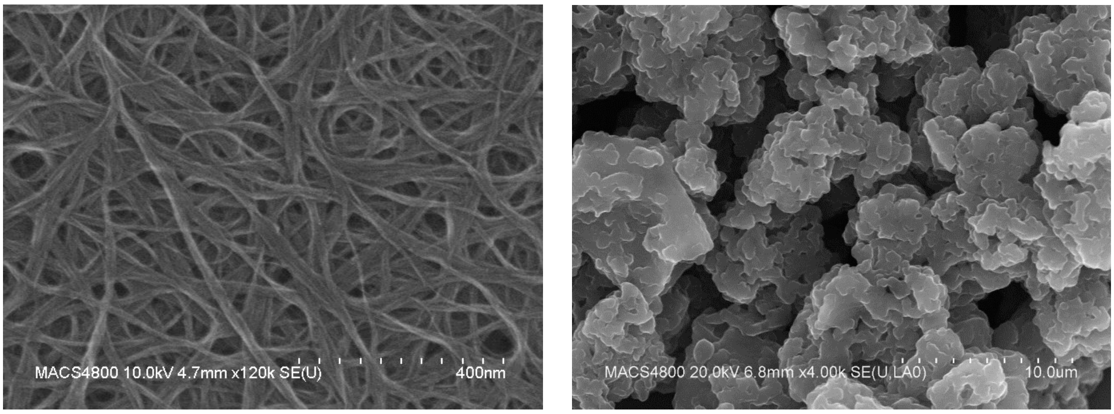

(**c**) (**d**)

Figure S2. SEM images of (**a**) carboxylic-SWCNTs; (**b**) sulfonated-SWCNTs; (**c**) hydroxyl-SWCNTs and (**d**) polyaniline.

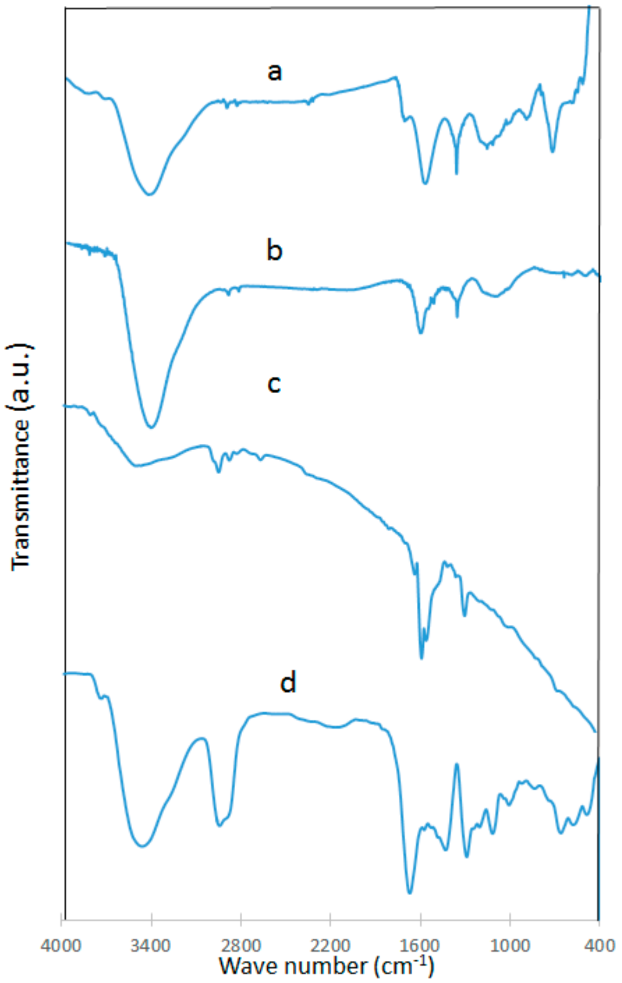

Figure S3. FTIR spectra. (**a**) carboxylic-SWCNTs; (**b**) sulfonated-SWCNTs; (**c**) hydroxyl-SWCNTs and (**d**) polyaniline.

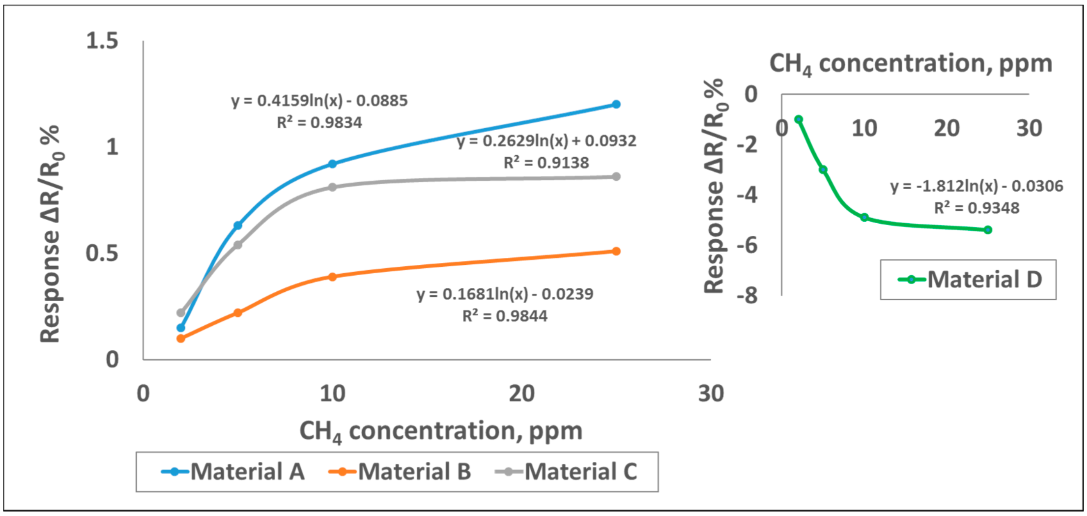

Figure S4. Calibration curves for methane using the four sensor materials (polyaniline in the inset). In each case, the calibration curve is from one of the four channels (see Table S1).

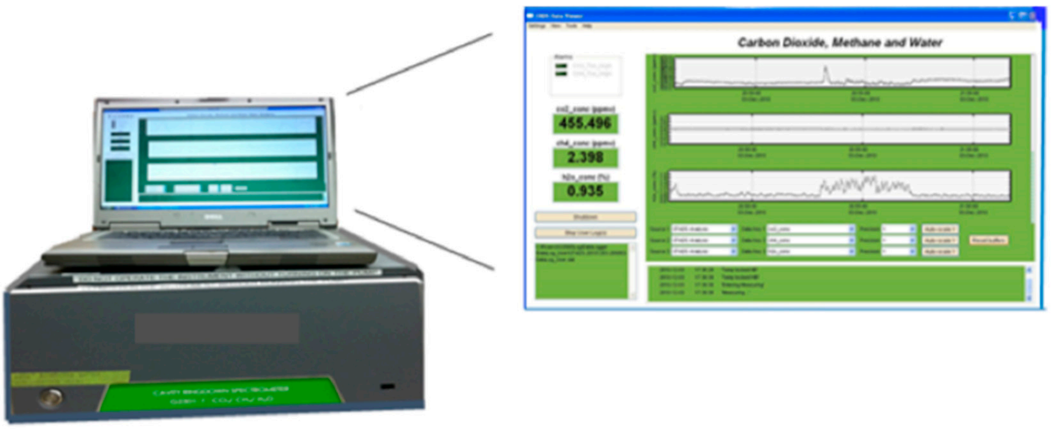

Figure S5. Picarro CDRS instrument.

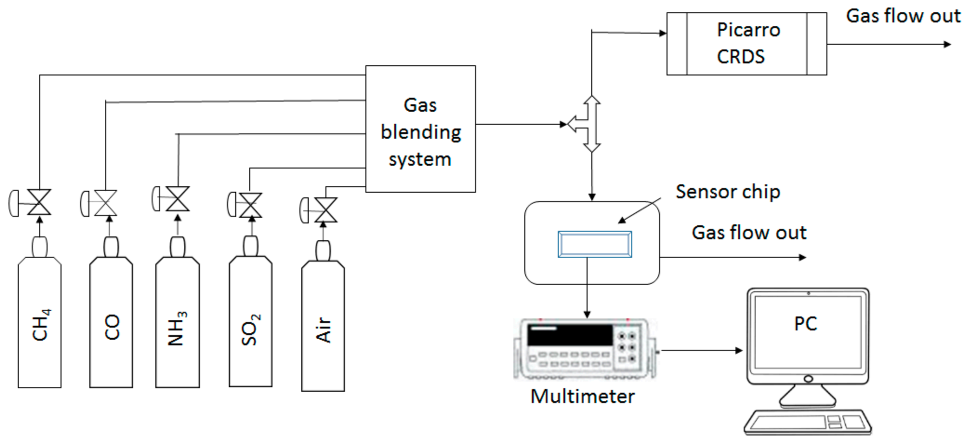

Figure S6. Schematic of the experimental set-up used for comparison of our chemiresistive sensor with the Picarro CRDS instrument.

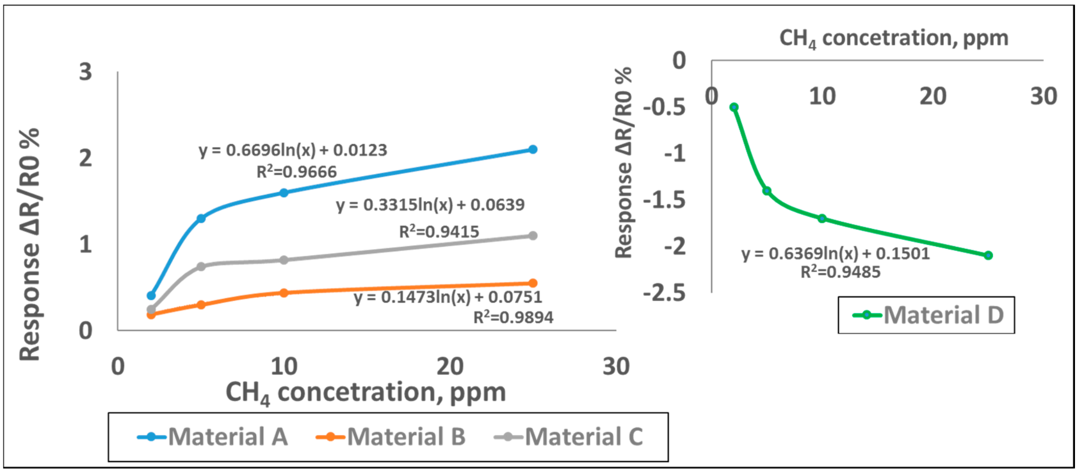

Figure S7. Calibration curves for methane from the smartphone sensor. One representative channel for each material from Table S1 is shown.
